# Advances in mt-tRNA Mutation-Caused Mitochondrial Disease Modeling: Patients’ Brain in a Dish

**DOI:** 10.3389/fgene.2020.610764

**Published:** 2021-01-12

**Authors:** Suleva Povea-Cabello, Marina Villanueva-Paz, Juan M. Suárez-Rivero, Mónica Álvarez-Córdoba, Irene Villalón-García, Marta Talaverón-Rey, Alejandra Suárez-Carrillo, Manuel Munuera-Cabeza, José A. Sánchez-Alcázar

**Affiliations:** ^1^Centro Andaluz de Biología del Desarrollo, Universidad Pablo de Olavide and Centro de Investigación Biomédica en Red: Enfermedades Raras, Instituto de Salud Carlos III, Seville, Spain; ^2^Instituto de Investigación Biomédica de Málaga, Departamento de Farmacología y Pediatría, Facultad de Medicina, Universidad de Málaga, Málaga, Spain

**Keywords:** mitochondrial diseases, mtDNA, disease modeling, direct reprogramming, induced neurons

## Abstract

Mitochondrial diseases are a heterogeneous group of rare genetic disorders that can be caused by mutations in nuclear (nDNA) or mitochondrial DNA (mtDNA). Mutations in mtDNA are associated with several maternally inherited genetic diseases, with mitochondrial dysfunction as a main pathological feature. These diseases, although frequently multisystemic, mainly affect organs that require large amounts of energy such as the brain and the skeletal muscle. In contrast to the difficulty of obtaining neuronal and muscle cell models, the development of induced pluripotent stem cells (iPSCs) has shed light on the study of mitochondrial diseases. However, it is still a challenge to obtain an appropriate cellular model in order to find new therapeutic options for people suffering from these diseases. In this review, we deepen the knowledge in the current models for the most studied mt-tRNA mutation-caused mitochondrial diseases, MELAS (mitochondrial encephalomyopathy, lactic acidosis, and stroke-like episodes) and MERRF (myoclonic epilepsy with ragged red fibers) syndromes, and their therapeutic management. In particular, we will discuss the development of a novel model for mitochondrial disease research that consists of induced neurons (iNs) generated by direct reprogramming of fibroblasts derived from patients suffering from MERRF syndrome. We hypothesize that iNs will be helpful for mitochondrial disease modeling, since they could mimic patient’s neuron pathophysiology and give us the opportunity to correct the alterations in one of the most affected cellular types in these disorders.

## Introduction: Mitochondria, the Powerhouses of the Cell

Mitochondria are small, mobile, and plastic organelles located in the cytoplasm of most eukaryotic cells. These organelles are responsible for important cellular processes, such as regulation of apoptosis, calcium homeostasis, and reactive oxygen species (ROS) production. However, the main function of mitochondria is energy production through oxidative phosphorylation (OXPHOS), which takes place in the mitochondrial respiratory chain (MRC) (Russell and Turnbull, 2014). Mitochondria contain their own DNA, the mitochondrial DNA (mtDNA), which is circular and double stranded. The mitochondrial genome consists of 16,569 nucleotide pairs that encode 13 proteins, two ribosomal RNA components, and 22 transfer RNAs (tRNAs) (Alberts et al., 2002). Regarding mitochondrial structure, these organelles are composed by two membranes, the inner and the outer mitochondrial membranes (IMMs and OMMs, respectively) that delimit two main compartments: the internal matrix and the intermembrane space. The IMM contains many folds named cristae that protrude into the matrix and enlarge the IMM surface. This membrane can be subdivided into two compartments, the inner boundary membrane (IBM) and the cristae membrane (CM), that are connected *via* cristae junctions. Albeit IMM is considered a continuous membrane, lateral diffusion of membrane proteins is restricted and IBM and CM exhibit an asymmetric protein distribution. This heterogeneity is important for efficient OXPHOS, mitochondrial biogenesis, and remodeling (Wollweber et al., 2017; Busch, 2020). Although mainly separated, the inner and outer mitochondrial membranes are partially connected *via* contact sites that are involved in cristae organization (Bulthuis et al., 2019). Since the OMM is more permeable than the IMM, containing many copies of the transport protein porin, the intermembrane space composition is equivalent to the cytosolic one. However, due to the presence of cardiolipin, the IMM is specially impermeable to ions and it is selectively permeable to small molecules required by matrix enzymes, thanks to the presence of several transport proteins (Alberts et al., 2002).

For producing energy, mitochondria rely on the MRC components that are five multiprotein complexes whose polypeptides are encoded by mtDNA and nuclear DNA (nDNA), the latter contributing the most. For that reason, mitochondrial function relies on both genomes (Alberts et al., 2002). Four of these components (Complexes I–IV) are involved in electron transfer and proton pumping across the IMM, which generate an electrochemical proton gradient responsible for the mitochondrial membrane potential (ΔΨm), a marker of mitochondrial health. The flux of protons into the matrix through the ATP synthase (Complex V) drives ATP synthesis (Alberts et al., 2002). There is a current consensus about a higher level of organization of these components that are arranged in supercomplexes (SCs) (Brzezinski, 2020) that exist in a wide variety of stoichiometries (Cogliati et al., 2016). SC assembly and stability are determined by mitochondrial cristae shape since knockdown of OPA1, a protein that plays a key role in mitochondrial fusion, impairs SC formation (Cogliati et al., 2013; Jang and Javadov, 2020). In addition, cellular pathways such as the unfolding protein response (UPR) are involved in cristae density and SC assembly, improving mitochondrial respiratory function under nutrient stress conditions (Balsa et al., 2019). Thus, this higher level of organization is dynamic and adapts to the energetic status of the cell. Structural and modeling studies indicate that SC assembly facilitates electron transfer, prevents protein aggregation, and preserves the structural organization of MRC components (Jang and Javadov, 2020). However, despite extensive studies, SC functional relevance remains unknown (Brzezinski, 2020).

Defects in mitochondrial function have been linked not only to genetic mitochondrial diseases but also to cardiovascular diseases (Murphy et al., 2016) and neurodegenerative disorders such as Huntington’s and Parkinson’s diseases (Zeviani and Carelli, 2003; Martin-Jimenez et al., 2020).

## Mitochondrial Diseases

Mitochondrial diseases are a heterogeneous group of rare genetic disorders caused by a partial or total dysfunction of mitochondria. These illnesses can be caused by mutations in nDNA or mtDNA. These mutations affect not only genes encoding for MRC components but also those that are involved in protein translation and assembly, mtDNA stability, as well as mutations in those nDNA-encoded proteins involved in the maintenance of mitochondrial nucleotide pools, nucleotide transport, mtDNA replication, RNA transcription, and mitochondrial dynamics (Schon et al., 2012; Chapman et al., 2020). Mitochondrial diseases are clinically heterogeneous; they may occur at any age, and patients manifest a wide variety of symptoms (Gorman et al., 2016). However, all of them share morphological and biochemical features. As a consequence of the MRC deficiency, cells manifest a reduced enzymatic function of MRC components, a reduction in oxygen consumption and ATP synthesis, and a ROS overproduction. In the case of patients, they suffer from lactic acidosis and elevated pyruvate levels in serum at rest and, specially, after moderate exercise. Additionally, patients’ muscle biopsies usually show ragged red fibers that reflect the proliferation of OXPHOS-defective mitochondria (Schon et al., 2012).

### Mitochondrial Diseases Caused by Mutations in Mitochondrial Transfer RNAs

Mutations in mtDNA are associated with a wide variety of maternally inherited genetic disorders that provoke mitochondrial dysfunction (Kirino and Suzuki, 2005). Since 1988, when the first mtDNA mutations were identified (Holt et al., 1988; Wallace et al., 1988; Zeviani et al., 1988), more than 400 pathogenic mutations related to specific and non-specific diseases have been characterized (Lott et al., 2013). Among them, some frequent mitochondrial disorders caused by a point mutation in mtDNA are MELAS and MERRF syndromes. In most of the cases, MELAS syndrome is caused by a transition from adenine to guanine in the position 3243 in the mt-tRNA^Leu(UUR)^ (MT-TL1) gene (Schon et al., 2012). In the case of MERRF syndrome, the m.8344A > G mutation in the mt-tRNA^Lys^ (MT-TK) gene is the most frequently associated with the disease (Shoffner et al., 1990; Yoneda et al., 1990; Kirino and Suzuki, 2005).

Pathogenic mutations in mt-tRNA affect mtDNA translation, causing a defect in protein synthesis and decreasing MRC complex function (Brisca et al., 2015). In the case of MELAS syndrome, the m.3243A > G mutation affects mt-tRNA structure stabilization, methylation, aminoacylation, and triplet recognition (Finsterer, 2007). This mutation causes a specific defect of UUG-rich gene translation such as ND6 gene that encodes a subunit of the NADH-coenzyme Q reductase complex, also known as Complex I. This translation defect results in specific Complex I deficiency due to a reduction in the synthesis of ND6 subunit that is characteristic of the MELAS syndrome (Kirino and Suzuki, 2005). On the other hand, m.8344A > G mutation, which accounts for the MERRF syndrome, affects both AAA and AAG codon translation, causing a defect of whole mitochondrial protein synthesis. This fact could explain some of the different symptoms that are associated with these two different diseases (Kirino and Suzuki, 2005).

Due to the multicopy nature of mtDNA, these mutations can be homoplasmic or heteroplasmic. Thus, MELAS and MERRF syndromes are heteroplasmic, which means that mutant and wild-type mtDNA copies coexist within the same cell. This feature is associated with the severity of the symptoms and hinders disease prognosis. In fact, it is thought that there is a threshold from which biochemical alterations are apparent (Zeviani and Carelli, 2007). This genetic heterogeneity gives rise to a wide variety of symptoms of diverse severity among patients. Both MELAS and MERRF syndromes are associated with neurological symptoms. MELAS syndrome affects several organs, and some of its manifestations include stroke-like episodes, dementia, epilepsy, lactic acidemia, myopathy, recurrent headaches, hearing impairment, diabetes, and short stature (El-Hattab et al., 2015). Stroke-like episodes are one of the main features of this syndrome, and patients’ brain MRIs usually show multiple stroke-like lesions in both occipital and temporoparietal areas (Angelini, 2014; El-Hattab et al., 2015). In the case of MERRF syndrome, the first symptom is usually myoclonus that is followed by generalized epilepsy, ataxia, weakness, and dementia. Other findings are hearing loss, short stature, optic atrophy, and cardiomyopathy (DiMauro, 2015).

### Therapeutic Management of Mitochondrial Diseases

The development of useful therapies for mitochondrial diseases is challenging due to the difficulty of correcting the lack or dysfunction of essential mitochondrial proteins, the phenotypical heterogeneity of the diseases, and multisystem alteration. Furthermore, the brain, one of the most affected organs, is difficult to reach by potential therapies because it is protected by the blood–brain barrier. For those reasons, there are no effective treatments available for mitochondrial diseases and management of these diseases is mainly symptomatic. Still, several strategies that are summarized in this review (Viscomi and Zeviani, 2020) have been developed.

Pharmacological treatment options are generally focused on targeting cellular pathways, such as mitochondrial biogenesis or autophagy, or preventing oxidative damage. For these reasons, AMP-activated protein kinase (AMPK) and mammalian target of rapamycin complex 1 (mTORC1) signaling have been the main targets of these strategies. Some of the most known drugs for treating mitochondrial diseases are rapamycin and its analogs (rapalogs) that inhibit mTORC1. Rapamycin and/or rapalogs have been demonstrated to improve the symptoms of some patients (Sage-Schwaede et al., 2019), but individual cases should be evaluated and long-term side effects remain unknown (Viscomi and Zeviani, 2020). Another common compound that is usually used for mitochondrial disease treatment is coenzyme Q_10_ (CoQ_10_), an essential component in the mitochondrial electron transport and antioxidant in cell membranes (Santa, 2010). However, improvement of symptoms under CoQ_10_ treatment has been variable, and no sustained clinical benefits have been reported (Santa, 2010; Gorman et al., 2016). The activation of mitochondrial biogenesis through AMPK is another common therapeutic option for these diseases. 5-Aminoimidazole-4-carboxamide-1-β-D-ribofuranoside (AICAR), an AMP analog that activates AMPK, has been demonstrated to ameliorate the clinical phenotype in mouse models of mitochondrial diseases (Viscomi and Zeviani, 2020), but it is still not clarified if all tissues will benefit from this treatment in the long term (Suomalainen and Battersby, 2018).

Particularly in mtDNA mutations, several supplements as antioxidants and cofactors are being used. In MELAS syndrome, these treatments are L-arginine, citrulline, CoQ_10_, creatine monohydrate, and L-carnitine (El-Hattab et al., 2015). In addition, oral taurine supplementation has been demonstrated to reduce the recurrence of stroke-like episodes and increase taurine modification in mt-tRNA^Leu(UUR)^ (Ohsawa et al., 2019). Moreover, idebenone, a CoQ_10_ analog, has shown promising results alone (Ikejiri et al., 1996) or in combination with riboflavin (Napolitano et al., 2000) when used in concrete patients. In Leber’s hereditary optic neuropathy (LHON), a mitochondrial disease commonly caused by a primary homoplasmic mutation in mtDNA (Meyerson et al., 2015), orphan designation (EU/3/07/434) was granted by the European Medicines Agency (EMA) for idebenone on 2007, and recently, commercialization of idebenone (Raxone) has been authorized by EMA for the treatment of visual impairment in adolescent and adult patients with this disease (EMA/480039/2015). In the case of MERRF syndrome, CoQ_10_, idebenone, or L-carnitine is frequently prescribed. Conventional anticonvulsant drugs, such as levetiracetam, are also used to treat seizures (DiMauro and Hirano, 1993; Finsterer, 2019). However, none of these treatments has demonstrated enough effectiveness and only partially ameliorate some symptoms.

Given the diversity of mutations and the different therapeutic options, a personalized therapeutic approach is required in mitochondrial diseases. For this reason, the development of cellular models derived from patients (discussed below) can be useful for both the evaluation of new drugs and the repositioning of existing ones.

Gene therapy is a promising alternative for treating mitochondrial diseases. Since pathogenic mtDNA mutations are usually heteroplasmic, reducing mutational load can be used as a therapeutic approach. There are several tools that could target mtDNA, but only two of them have been demonstrated to be successful: zinc-finger nucleases (ZFNs) and transcription activator-like effector nucleases (TALENs). Both tools are delivered into mitochondria using a mitochondria localization signal, and they selectively target mtDNA sequences to create double-strand breaks (Reddy et al., 2020).

Zinc-finger nucleases have been demonstrated to reduce mutant mtDNA and consequently restore mitochondrial respiratory function in cytoplasmic hybrid (cybrid) cell models (Minczuk et al., 2008; Gammage et al., 2014). In addition, this tool has been able to specifically eliminate mutant mtDNA in the cardiac tissue of a mitochondrial disease mouse model (Gammage et al., 2018). ZFNs are small and, due to their similarity to mammalian transcription factors, they are thought to have low immunogenic properties. However, they are complex, expensive, and exhibit lower specificity and efficiency than TALENs (Reddy et al., 2020). TALENs have been widely used for genetic manipulation in different organisms. This tool has been demonstrated to reduce mutant mtDNA load and improve the pathophysiology in a cellular model of MERRF syndrome (Hashimoto et al., 2015) as well as to eliminate the m.3243A > G mutation in MELAS iPSCs and porcine oocytes (Yang et al., 2018). In addition, TALENs have been able to reduce mutant mtDNA load in a mouse model harboring a mutation in a mt-tRNA, reverting disease-related phenotypes (Bacman et al., 2018). These gene therapy tools could be used together with other therapies aiming the elimination or prevention of pathogenic mtDNA transfer from mother to child (Nissanka and Moraes, 2020).

Regarding prevention of these diseases, the only option available is transferring embryos below the threshold of clinical expression in order to avoid or at least reduce the risk of transmission of mtDNA mutations. The selection of these embryos is based on preimplantation genetic diagnosis (PGD) (Gorman et al., 2016). In addition, there is a new strategy, the mitochondrial donation, that consists of the substitution of mutant maternal mitochondria using enucleated donor oocytes (Gorman et al., 2016). However, this technique has raised ethical issues and remains controversial (Saxena et al., 2018).

## Common Models for Mitochondrial Disease Study

Clinical research in mitochondrial diseases has been traditionally carried out in many patients worldwide. There are many articles regarding these disorders that are in fact case reports. These studies have provided valuable information about these diseases such as the clinical hallmarks, the mutations that cause the disease, the onset, and the effects of the available treatments and supplements. In addition, they help to infer the pathophysiological mechanisms underlying these diseases.

**FIGURE 1:**
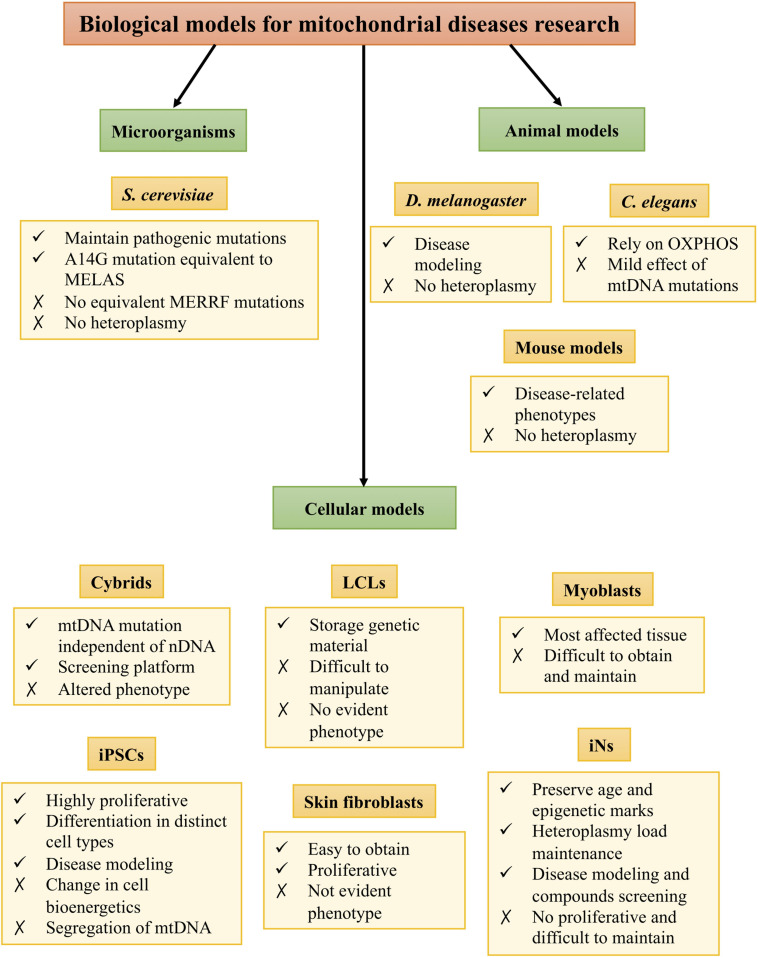
Biological models for mitochondrial disease research.

However, experimental research has been hindered by the lack of proper biological models. These models are necessary for understanding the pathophysiology of the diseases as well as for finding new therapeutic targets. Despite the difficulties to mimic the repercussions of mtDNA mutations, there are several models to study mitochondrial diseases, such as microorganism, animal, and cellular models (Figure 1).

### Microorganism Models

*Saccharomyces cerevisiae* is the most used microorganism model to study mitochondrial diseases. Mitochondrial functions are highly conserved between humans and *S. cerevisiae*, and this microorganism maintains pathogenic mutations that lead to mitochondrial dysfunction in humans. Therefore, yeasts are a good model to study mitochondrial diseases, providing insight into both physiological and pathophysiological processes (Baile and Claypool, 2013). The *S. cerevisiae* strain harboring the A14G mutation is equivalent to the human m.3243A > G mutation that causes MELAS syndrome. This model has been demonstrated to be suitable for compound screening (Garrido-Maraver et al., 2012). In the case of MERRF syndrome, there are no yeast strains with an equivalent mutation. However, the SLM3 gene in yeast is homologous to human TRMU, an enzyme involved in mt-tRNA wobble position thiolation. Mutations in SLM3 gene cause phenotypic features similar to those of m.8344A > G mutation, and consequently, yeasts harboring a defect in that gene have been used to study MERRF syndrome (Umeda et al., 2005). However, they do not maintain these mutations in a heteroplasmic state, so this aspect cannot be assessed using this model (Rinaldi et al., 2010).

### Animal Models

To deepen the knowledge in the consequences of mtDNA mutations as well as develop new therapeutic strategies for mitochondrial diseases, animal models are necessary. However, there are only a few animal models for studying mitochondrial diseases, and none of them is specific for MELAS and MERRF syndromes.

The fruit fly *Drosophila melanogaster* is an excellent model organism for studying molecular mechanisms of human diseases and has been used for modeling neurodegenerative diseases such as Alzheimer’s and Parkinson’s diseases as well as for drug discovery (Pandey and Nichols, 2011). In the case of mitochondrial diseases, there are *Drosophila* mutants of MRC components that show neurodegeneration, motor dysfunction, increased ROS production, and abnormal mitochondrial morphology (Liu et al., 2007; Mast et al., 2008), as well as mutants of mtDNA, such as a model of Leigh syndrome (Celotto et al., 2006). However, currently, it is not possible to generate a *Drosophila* mutant with intermediate heteroplasmy for studying MELAS and MERRF syndromes (Palladino, 2010).

Mitochondrial composition, function, and mtDNA are well conserved between *Caenorhabditis elegans* and humans (Falk et al., 2009). *C. elegans* growth and development are energy-dependent, and the main ATP source for this organism is the MRC, which makes it interesting for studying mitochondrial diseases. In fact, there are worm strains that harbor mutations in MRC subunits encoded by nDNA (Ishii et al., 1998; Kayser et al., 2001; Tsang et al., 2001). However, mtDNA mutations have a mild effect in *C. elegans* (Tsang and Lemire, 2003), and they do not mimic the pathophysiological features of mtDNA mutation-caused mitochondrial diseases.

Mammalian models are necessary for studying pathogenesis or tissue-specific alterations that cannot be addressed using other models. Several murine models for OXPHOS deficiencies caused by mutations in nuclear genes encoding for MRC complexes, regulatory factors, and other components required for mitochondrial function have been created by transgenesis (Iommarini et al., 2015; Torraco et al., 2015). However, this is not the case for diseases caused by mutations in mtDNA, whose modeling is technically difficult (Tyynismaa and Suomalainen, 2009). Different mutagenesis methods to generate mutations in mouse mtDNA have been described, and these mutations can be transferred from cultured cells into mice, thereby creating transmitochondrial mice (mito-mice) (Inoue et al., 2000; Nakada et al., 2001; Fan et al., 2008; Kauppila et al., 2016). For example, a mouse model of LHON has been generated by introducing the equivalent of the human ND6 G14600A P25L mutation in homoplasmy into the mouse. Mutant mice showed a reduction in retinal function and neuronal accumulation of abnormal mitochondria among other events probably due to partial Complex I defects and increased ROS production (Lin et al., 2012). However, the generation of a mouse model with pathological heteroplasmic mtDNA mutations has proved challenging due to the multicopy nature of the mitochondrial genome. Furthermore, the transfection of plasmids or modified mtDNA into mouse mitochondria has not been successful (McGregor et al., 2001).

### Cellular Models

Due to the lack of proper animal models for studying these diseases, cellular models have been extensively used in order to study MELAS and MERRF syndromes. Among them, we can find transmitochondrial cybrids, human B lymphoblastoid cell lines (LCLs), patient-derived cells such as myoblasts and dermal fibroblasts, and induced pluripotent stem cells (iPSCs) (Hu et al., 2019).

Transmitochondrial cybrids are generated by fusing enucleated cells that harbor wild-type or mutated mtDNA with ρ(0) cells, in which endogenous mtDNA has been depleted. For that reason, they are very useful for studying mtDNA mutations excluding the influence of nDNA variability (Vithayathil et al., 2012; Wilkins et al., 2014), and they have been used for studying MELAS and MERRF syndromes (Cotan et al., 2011; De la Mata et al., 2012; Garrido-Maraver et al., 2012; Villanueva-Paz et al., 2020). However, cybrid models show important limitations such as the need for a high mutational load to observe some pathophysiological features and the alteration in the cell behavior due to the loss of nDNA and mtDNA interactions (Dunbar et al., 1995).

Lymphoblastoid cell lines are generated by transformation of peripheral B lymphocytes and constitute a valuable source of mitochondria to study their function in mitochondrial diseases patients (Bourgeron et al., 1992). This model offers advantages such as the facility to obtain large quantities of lymphocytes and that they can be immortalized efficiently (Hu et al., 2019). For that reason, they have been used to study MERRF syndrome (Chang et al., 2013) and other mitochondrial diseases (Chin et al., 2018). Although this model is still the choice of storage for patients’ genetic material due to its low somatic mutation rate and ease of maintenance, LCLs have some limitations such as the presence of two different cellular stages and the different response in comparison with other cell types (Sie et al., 2009). Moreover, they are difficult to manipulate, and the phenotype is often not evident.

Myoblasts derived from patients’ biopsies are a very attractive model because they belong to one of the most affected tissues in these diseases. For that reason, they have been used to study the role of antioxidant enzymes and ATP levels in MELAS syndrome (Rusanen et al., 2000) and the distribution and expression of mutant mtDNAs in MERRF syndrome (Boulet et al., 1992). However, using myoblasts as a cellular model for mitochondrial diseases has several drawbacks. First, large quantities of proliferative myoblasts are difficult to isolate from a muscle tissue biopsy at later stages, so it is probable that more than one muscle biopsy is necessary to obtain enough cells for the analysis required. Moreover, primary myoblasts demand special conditions for optimal growth, and myoblast enrichment protocols are needed in order to obtain a pure cell culture.

Cultured fibroblasts are other patient-derived cells that have been a useful tool to study mitochondrial diseases. This model offers numerous advantages, since it is easy to obtain from a little invasive process such as a skin biopsy. In addition, fibroblast cultures are highly proliferative and provide a renewable source of cells (Hu et al., 2019). This model has been and still is widely used for studying cellular pathophysiology and as a screening tool for MELAS and MERRF syndromes (Wu et al., 2010; Cotan et al., 2011; De la Mata et al., 2012; Garrido-Maraver et al., 2015; Hayashi and Cortopassi, 2015; Villanueva-Paz et al., 2020) and other mitochondrial diseases. Nevertheless, it has some drawbacks as a cellular model for these diseases. First, they rely on glycolytic metabolism for energy production; therefore, they are not much vulnerable to energy-dependent defects resulting from mitochondrial dysfunction. In addition, they are difficult to maintain in culture and sometimes it is challenging to observe pathophysiological alterations, especially when the heteroplasmy load is low.

The most affected cell types in these diseases are the brain and the skeletal muscle cells, since they have a huge mitochondrial density due to high energy requirements and, consequently, they are more vulnerable to defects caused by mitochondrial dysfunction (DiMauro, 2007). In fact, neuron alteration is such that MELAS and MERRF syndromes are considered neurodegenerative mitochondriopathies in which there is neuronal cell death. MELAS neurodegeneration usually involves cortical territories in occipital and temporoparietal areas, as well as neurons in the Purkinje layer; meanwhile, in MERRF syndrome, the most affected areas are the Purkinje layer and dentate nucleus (Swerdlow, 2009).

The appearance of iPSCs in 2006 (Takahashi and Yamanaka, 2006) has led to numerous possibilities in the field of disease modeling. In fact, it has been a great step forward in the study of neurodegenerative diseases (Ebert et al., 2009; Lee et al., 2009; Kondo et al., 2013), as well as mitochondrial diseases (Cherry et al., 2013; Kodaira et al., 2015; Chou et al., 2016; Zhang et al., 2016; Inak et al., 2017; Lorenz et al., 2017; Yang et al., 2018). These iPSCs can be differentiated into somatic cell types such as neurons, which allow the study of these diseases in one of the most affected cellular types.

In the case of MERRF syndrome, neural progenitor cells (NPCs) derived from iPSCs have been demonstrated to reproduce pathophysiological features previously observed in other models of the disease, such as an impaired mitochondrial respiration, increased ROS production, altered antioxidant enzyme expression, as well as a fragmented mitochondrial network (Chou et al., 2016). Regarding MELAS syndrome, iPSC-derived neurons have made possible the study of not only common pathophysiological alterations but also neuron-specific alterations in the disease. For instance, in this work (Klein Gunnewiek et al., 2020), MELAS iPSC-derived neurons harboring a high heteroplasmy load showed lower dendrite complexity compared to control and low heteroplasmy neurons. These neurons also exhibited a reduced synaptic density, axonal mitochondrial abundance, and frequency of spontaneous excitatory activity, as well as an impaired neuronal network activity and synchronicity.

In addition, iPSC-derived neurons from mitochondrial disease patients can show different pathophysiological characteristics than parental patient-derived cells. For example, Hämäläinen et al. (2013) observed that parental MELAS fibroblast lines with intermediate heteroplasmy levels showed a combined deficiency of mtDNA-encoded Complexes I, III, and IV subunits; meanwhile, MELAS iPSC-derived neurons manifested a remarkable CI deficiency, which is typical for MELAS patient tissues and commonly reported upon mitochondria-associated neurodegeneration.

**FIGURE 2:**
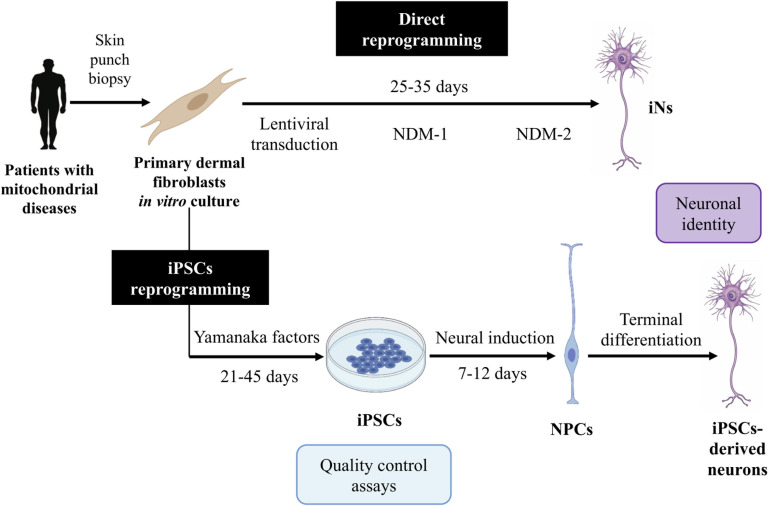
Induced neurons (iNs) and induced pluripotent stem cell (iPSC)-derived neuron generation. This figure summarizes the protocols for the generation of iNs and iPSC-derived neurons. For iN generation, following lentiviral transduction of neural transcription factors, cells are exposed to a neural differentiation medium (NDM) I containing specific small molecules and growth factors and then to an NDM II containing only the growth factors. iNs are obtained at 25–35 days post-infection (DPI). For iPSC-derived neuron generation, fibroblasts are transduced with Yamanaka factors to generate iPSCs. These iPSCs are converted into neural progenitor cells (NPCs) by defined factors, and these are then differentiated into the desired neuron subtype.

Reprogramming into iPSCs offers several advantages, since iPSCs can be cultured and a large quantity of starting material can be obtained. However, the protocol is complex, expensive, and time-consuming (Dolmetsch and Geschwind, 2011; Figure 2). Furthermore, this technique results in some disadvantages related to their use in mitochondrial disease research.

Several studies have demonstrated that reprogramming into iPSCs resets cellular age (Hsu et al., 2016), obtaining young neurons that could not show cell pathophysiology and, as a result, might not be useful to study neurodegenerative diseases. Furthermore, another study shows that this approach causes mitochondrial rejuvenation and an improvement in the cellular energy production capacity upon differentiation (Suhr et al., 2010). Another drawback would be that the use of these cells in cell replacement therapies is limited due to uncompleted differentiation and their propension to form tumors (Parmar and Jakobsson, 2011). In addition, analysis of mtDNA variants in iPSCs showed that low levels of potentially pathogenic mutations in the fibroblasts are revealed during reprogramming into iPSCs. Thus, mutant iPSCs are generated, causing a deleterious effect on their differentiated progeny and contributing to intra-person variability. This accumulation of mtDNA mutations can impact metabolic functions in iPSCs, hampering mitochondrial respiration (Kang et al., 2016; Perales-Clemente et al., 2016). These results highlight the need for monitoring mtDNA mutations and examining the metabolic status and quality of iPSCs intended for disease modeling or drug screening. Finally, reprogramming into iPSCs provokes an mtDNA segregation toward homoplasmy. In this way, after reprogramming, mutant and wild-type cells are obtained (Pickrell and Youle, 2013). These wild-type cells might be useful for cell therapy or as a syngeneic control, since they would be identical but will not express pathophysiological features. Therefore, the iPSC approach could be considered an autologous source of material suitable for cell therapy, without the risk of immune rejection (Smith et al., 2015). However, homoplasmic mutant iPSCs would not reflect patients’ cell pathophysiology, since they harbor the mutation in a heteroplasmic state. Thus, there are major limitations that we still have to overcome, and currently, iPSCs have no therapeutic use (Crow, 2019). All of the above reasons make necessary the generation of additional models for these diseases.

## New Model for Mitochondrial Disease Research: Induced Neurons Generated by Direct Reprogramming

Direct reprogramming of fibroblasts into induced neurons (iNs) was achieved for the first time in 2010 (Vierbuchen et al., 2010). Using a combination of proneural transcription factors such as Ascl1, Brn2, and Myt1l, Vierbuchen et al. (2010) achieved the conversion of embryonic and postnatal murine fibroblasts into functional neurons *in vitro*. One year later, several advances allowed the improvement of the technique and increased the conversion efficiency. One of them was the utilization of microRNAs (miRNAs), in concrete miR-9/9^∗^ and miR-124, that leads to the conversion of fibroblasts into neurons even without the overexpression of proneural factors. However, since the reprogramming efficiency was low, these miRNAs were combined with the expression of NeuroD2 factor, Ascl1 and Myt1l (Yoo et al., 2011). Alternatively, using NeuroD1 factor together with the overexpression of Ascl1, Brn2, and Myt1l, direct reprogramming of fibroblasts into iNs was achieved with high efficiency (Pang et al., 2011). Additionally, compound screening has allowed the usage of a combination of small molecules and neural growth factors that increase reprogramming efficiency (Ladewig et al., 2012; Pfisterer et al., 2016).

Later, Drouin-Ouellet et al. (2017a) described a barrier for adult fibroblast reprogramming, the REST (RE-1 silencing transcription factor) complex, and developed one single lentiviral vector for the conversion of human adult fibroblasts into iNs in a very efficient manner. These iNs expressed neuron-specific proteins and exhibited electrophysiological properties. A recent paper from this group describes that the addition of the miRNAs indicated above, together with the overexpression of Ascl1 and Brn2, and the REST complex silencing, support neuronal maturation (Birtele et al., 2019). This technique allows the generation of a pan-neuronal population, but there are other strategies for reprogramming into neuronal subtypes, such as dopaminergic (Caiazzo et al., 2011), motor (Son et al., 2011; Qin et al., 2018), and serotonergic neurons (Xu et al., 2016). These approaches are valuable for some diseases in which there is one specific neuronal subtype affected or even different neuron subtypes may express distinct disease-related phenotypes.

Direct reprogramming brings numerous advantages. First, the procedure is relatively simple and fast (Ladewig et al., 2013; Figure 2). In addition, iNs maintain the age (Mertens et al., 2015) and the epigenetic marks of the donor (Horvath, 2013; Huh et al., 2016), which make them excellent models to study neurodegenerative diseases such as mitochondrial disorders. Furthermore, iNs have demonstrated to not cause tumorigenic processes after *in vivo* reprogramming (Torper et al., 2013), so they might be a promising tool for cell therapy. Thus, iN generation from patients’ fibroblasts is thought to be a useful approach for studying the pathogenesis of these diseases.

One of the main challenges of direct reprogramming is reaching a high conversion efficiency. It is defined as the percentage of iNs obtained over the number of cells plated for conversion and can be very variable depending on the starting cells and the protocol used. The first approaches using direct reprogramming obtained very poor conversion efficiencies (Yoo et al., 2011; Ladewig et al., 2013), but the single vector-based approach developed by Drouin-Ouellet et al. (2017a; 2017b) and Shrigley et al. (2018) can generate very high yields of iNs. However, it is also necessary to reach a high percentage of purity, which is the number of iNs in the final population over the cells remaining in the plate. These two parameters are crucial since neurons are post-mitotic cells not able to further expand.

In addition, maintaining iNs in long-term cultures is difficult and expensive, since cell death can be observed from 30 days post-infection (DPI). This fact may hinder electrophysiological characterization of iNs, since the earliest time point when spontaneous action potentials have been detected is at 80–100 DPI (Drouin-Ouellet et al., 2017a). Moreover, iNs tend to form clusters during the reprogramming process, hampering isolation of individual cells for further analysis.

To our knowledge, the first work that uses iNs for mitochondrial disease study comes from our group. Following the protocol established for generating iNs using a single lentiviral vector (Shrigley et al., 2018), we successfully generated iNs from two MERRF patient-derived fibroblasts harboring the m.8344A > G mutation (Villanueva-Paz et al., 2019). The proportion of this mutation in the fibroblast cultures was 66% in MERRF 1 and 33% in MERRF 2 fibroblasts, and we demonstrated that iNs maintained these proportions after reprogramming. The maintenance of the heteroplasmy load is crucial if iNs are going to be used for disease modeling or as a screening platform. In addition, this fact makes direct reprogramming a more suitable approach for studying mitochondrial diseases than iPSC generation because, in the latter case, mtDNA segregation and a tendency to homoplasmy have been observed (Pickrell and Youle, 2013).

In this work (Villanueva-Paz et al., 2019), the presence of bouton-like structures and spine-like protrusions was observed in both control and MERRF iNs, suggesting neuronal maturation. Furthermore, in another paper from our group, we performed electrophysiological recordings, and iNs showed electrophysiological properties at 60–80 DPI (Villanueva-Paz et al., 2020). The presence of neuronal maturation markers and functional properties suggest that iNs are behaving like neurons and make them a good candidate for mimicking the alterations happening in the patients’ brain. We also characterized the iN areas, perimeters, neurite features, as well as mitochondrial morphology, and we observed differences between control and MERRF iNs, indicating that the maintenance of the mutational load was affecting these features (Villanueva-Paz et al., 2019).

In this regard, MERRF iNs also showed pathophysiological features that have been described in other models of the disease such as fibroblasts, cybrids (De la Mata et al., 2012; Villanueva-Paz et al., 2020), and NPCs derived from MERRF iPSCs (Chou et al., 2016). For instance, they showed a reduced ΔΨm, a ROS overproduction, a disrupted autophagy flux, and an increased mitophagy (Villanueva-Paz et al., 2019). This fact makes them a good model for studying the cellular alterations happening in one of the most affected cell types in the disease. MERRF iNs were also suitable for performing other experiments such as the assessment of cellular bioenergetics using an extracellular flux analyzer. In these experiments, MERRF iNs showed alterations in the bioenergetic status such as reduced basal and maximal respirations, spare respiratory capacity, and ATP production (Villanueva-Paz et al., 2019). Some of these alterations were able to be rescued by Guttaquinon CoQ_10_ (a water-soluble derivative of CoQ_10_) treatment, demonstrating that iNs are not only suitable for disease modeling but also for compound screening (Villanueva-Paz et al., 2020).

## Conclusion and Future Perspectives

The generation of mitochondrial disease patient-derived iNs is a very promising starting point for the advance in the study of these illnesses and the search for new treatments. This model has been demonstrated to have neuronal identity as well as to reproduce pathophysiological features of the diseases (Villanueva-Paz et al., 2019). Among these characteristics, they have been able to show mitochondrial dysfunction that is a hallmark in mitochondrial disorders and other neurodegenerative illnesses such as Parkinson’s or Huntington’s diseases (Zeviani and Carelli, 2003). The presence of these features in iNs is even more important due to the fact that they are the most affected cell types in neurodegenerative disorders. This model also brings other advantages for studying this kind of diseases, such as the maintenance of the age and the epigenetic marks of the donor (Horvath, 2013; Mertens et al., 2015; Huh et al., 2016). For those reasons, we think that iNs are very valuable for modeling diseases that are accompanied by mitochondrial dysfunction. An interesting further step in heteroplasmic mitochondrial disease modeling would be the establishment of 3-dimensional (3D) cultures, such as cerebral organoids (Amin and Pasca, 2018), in which iNs as the main cellular component would presumably be maintained in culture for prolonged periods while remaining viable and retaining their specific activities. This 3D model has already been generated using iPSCs derived from mitochondrial neurogastrointestinal encephalomyopathy (MNGIE) patients’ cells (Pacitti and Bax, 2018). In addition, iNs have been demonstrated to be suitable for compound screening, since bioenergetic status of MERRF iNs was able to be rescued by Guttaquinon CoQ_10_ treatment (Villanueva-Paz et al., 2020). However, we still have to go deeper into the characterization of these new models and their suitability for finding new therapeutic targets for mitochondrial diseases.

## Author Contributions

SP-C wrote the manuscript. JS-R, MÁ-C, IV-G, MT-R, AS-C, and MM-C contributed to the literature search. MV-P and JS-A revised the manuscript. All authors contributed to the article and approved the submitted version.

## Conflict of Interest

The authors declare that the research was conducted in the absence of any commercial or financial relationships that could be construed as a potential conflict of interest.

## Abbreviations

ΔΨ m: mitochondrial membrane potential
3D: 3-dimensional
CM: cristae membrane
CoQ_10_: coenzyme Q_10_
DPI: days post-infection
EMA: European Medicines Agency
IBM: inner boundary membrane
IMM: inner mitochondrial membrane
iNs: induced neurons
iPSCs: induced pluripotent stem cells
LCLs: lymphoblastoid cell lines
LHON: Leber’s hereditary optic neuropathy
MELAS: mitochondrial encephalomyopathy lactic acidosis and stroke-like episodes
MERRF: myoclonic epilepsy with ragged red fibers
miRNAs: micro RNAs
MNGIE: mitochondrial neurogastrointestinal encephalomyopathy
MRC: mitochondrial respiratory chain
mtDNA: mitochondrial DNA
NDM: neural differentiation medium
nDNA: nuclear DNA
NPCs: neural progenitor cells
OMM: outer mitochondrial membrane
OXPHOS: oxidative phosphorylation
PGD: preimplantation genetic diagnosis
REST: RE-1 silencing transcription factor
ROS: reactive oxygen species
SCs: supercomplexes
TALENs: transcription activator-like effector nucleases
tRNA: transfer RNA
UPR: unfolding protein response
ZFNs: zinc-finger nucleases.
